# Two-year outcome of an eye that underwent hyperopic LASIK following inadvertent myopic SMILE lenticule in situ implantation

**DOI:** 10.1186/s12886-019-1188-9

**Published:** 2019-08-08

**Authors:** Jing Zhao, Jianmin Shang, Lingling Niu, Haipeng Xu, Dong Yang, Yu Zhao, Dan Fu, Xingtao Zhou

**Affiliations:** Department of Ophthalmology, Eye and ENT Hospital of Fudan University, Key NHC Key Laboratory of Myopia; Laboratory of Myopia, Chinese Academy of Medical Sciences, 83 FenYang Road, Shanghai, 200031 China

**Keywords:** SMILE, Lenticule, Re-implantation, Refractive error

## Abstract

**Background:**

This report describes a case in which hyperopic femtosecond laser-assisted in situ keratomileusis (FS-LASIK) was performed following small incision lenticule extraction (SMILE) lenticule in situ implantation.

**Case presentation:**

The hyperopic left eye of a 46-year-old patient with refraction of + 7.75 diopters sphere (DS)/− 1.25 diopters cylinder (DC) × 5° and corrected distance visual acuity (CDVA) of 20/50 mistakenly underwent the SMILE procedure for myopic astigmatism (− 8.50 DS/− 1.50 DC × 175°) due to medical negligence. The extracted lenticule was subsequently re-implanted in situ. After 8 months, the left eye underwent FS-LASIK to correct hyperopia and astigmatism (+ 5.0 DS/− 0.75 DC × 100°). Two years after FS-LASIK, corneal tomography showed no ectasia and microscopy revealed transparent cornea. The left eye exhibited CDVA of 20/50 with refraction of − 0.75 DS/− 0.25 DC × 165°.

**Conclusions:**

SMILE lenticule in situ implantation offers a solution for corneal volume and thickness restoration. FS-LASIK provides feasible correction of refractive error following lenticule re-implantation. Future studies are needed for determining the effectiveness of the treatment.

## Background

Small incision lenticule extraction (SMILE) has been proposed as an alternative to laser-assisted in situ keratomileusis (LASIK) for treatment of myopia and astigmatism, as it causes fewer dry-eye symptoms and avoids flap-related complications [1–3]. Moreover, refractive lenticules extracted during the SMILE procedure can be used successfully for autologous or allogenic implantation to reverse myopic correction and treat presbyopia, hyperopia, keratoconus, and other corneal diseases [4–10]. Notably, it is rare for a patient with hyperopia to mistakenly undergo myopic SMILE. To the best of our knowledge, we present the first report of SMILE lenticule re-implantation in situ to restore corneal volume and thickness in a patient’s cornea, followed by LASIK to correct hyperopia and astigmatism after negligent refractive surgery.

## Case presentation

A 46-year-old man was transferred to the doctor (Prof. Xingtao Zhou) of our refractive surgery center after negligent refractive surgery. Only a portion of the preoperative information and intraoperative parameters were available. Preoperative ophthalmic examinations were normal, with the exception of hyperopia with astigmatism and amblyopia. Uncorrected distance visual acuity (UDVA) was 20/125 for both eyes. Manifest refraction was OD: + 7.25 diopters sphere (DS)/− 1.25 diopters cylinder (DC) × 10° with corrected distance visual acuity (CDVA) of 20/40, OS: + 7.75 DS/− 1.25 DC × 5° with CDVA of 20/50. The right eye was the dominant eye and the additional power for near reading was + 0.25 D. Corneal tomography was evaluated by a Scheimpflug camera (Pentacam; Oculus, Wetzlar, Germany). The respective preoperative central corneal thickness (CCT) and mean keratometry readings were 523 μm and 41.3 D in the right eye, whereas they were 517 μm and 41.4 D in the left eye. Intraocular pressure (IOP) was normal in both eyes.

The patient complained of vision fatigue at both far and near distances, and he was scheduled for bilateral femtosecond laser-assisted in situ keratomileusis (FS-LASIK) to treat hyperopia and astigmatism. The flap creation was first performed on the patient’s right eye using the VisuaMax femtosecond laser (Carl Zeiss Meditec AG, Jena, Germany). Then, a SMILE procedure to treat myopic astigmatism (refractive correction: -8.50 DS/− 1.50 DC × 175°) was mistakenly performed on the patient’s left eye with the same system, due to medical negligence. The attempted lenticule thickness was 134 μm, with cap thickness of 110 μm and optical zone of 6.1 mm. A 90° single side cut with a length of 2 mm was created in the superior position. When the patient was transferred to the excimer laser platform for stromal ablation on his right eye, the erroneous operation in the left eye was quickly recognized. Subsequently, stromal ablation for hyperopia and astigmatism were performed in the patient’s right eye, comprising an uneventful FS-LASIK procedure. The extracted lenticule of the left eye was temporarily maintained in balanced salt solution (BSS), and the patient was immediately transferred to the Dr. Zhou.

The extracted lenticule of the left eye was re-implanted in situ within 2 h. A lamellar dissector was inserted to gently release the pocket adhesions. The refractive lenticule was grasped with a forceps and partially folded, then inserted into the pocket gradually through the small incision. Thereafter, the lamellar dissector was used to spread the lenticule; each edge of the lenticule was carefully flattened and smoothened with a spatula. To remove the striae, the cap was hydrostretched with sponge swabs soaked in BBS. Each step of the treatment was carefully evaluated and adjusted to enable proper positioning of the refractive lenticule. However, the surgeon inserted the lenticule without knowledge of the anterior or posterior surface, and without correctly aligning the axis of astigmatism, because there was no mark on the lenticule.

**Fig. 1:**
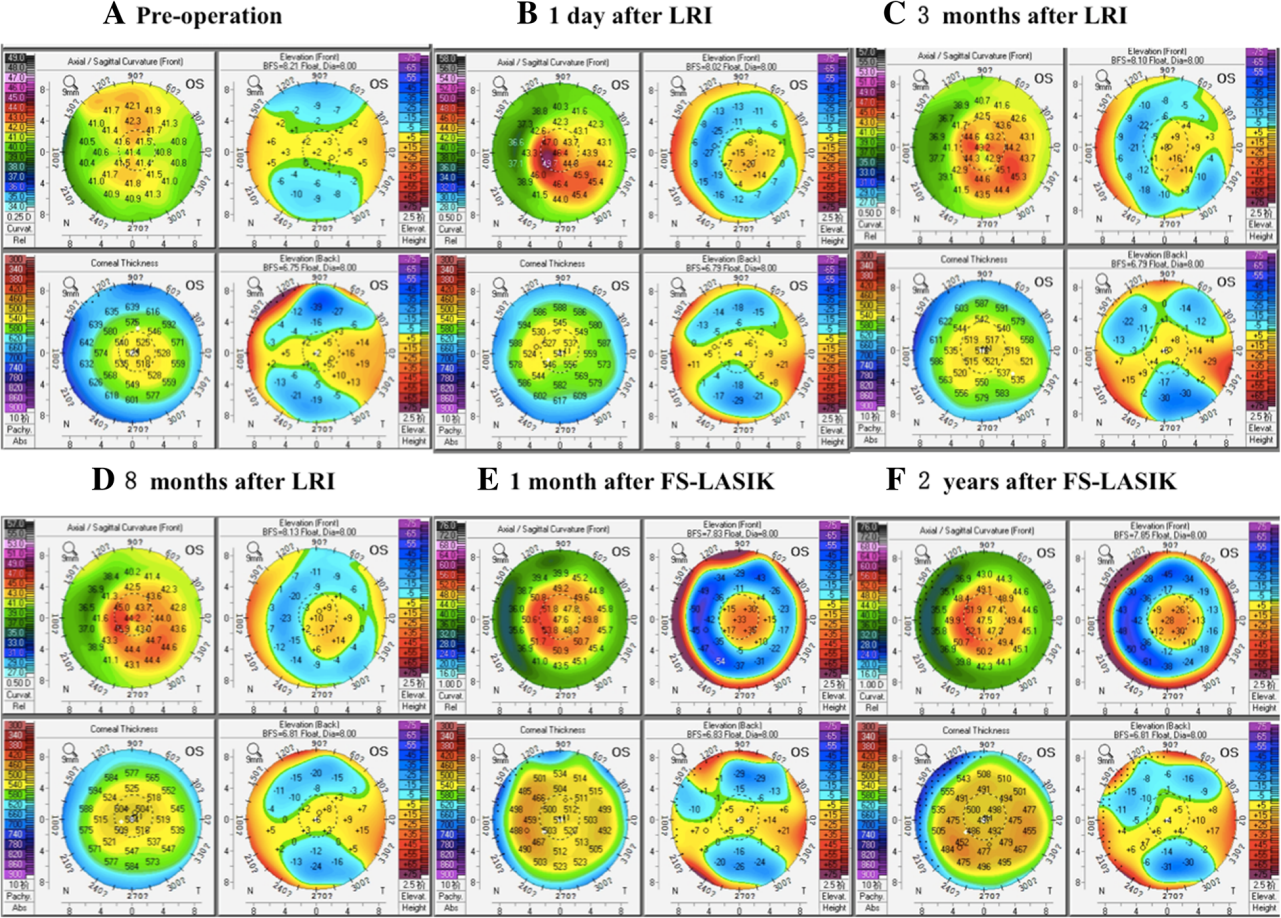
Corneal topographic images before operation, after refractive lenticule re-implantation (LRI), and after femtosecond laser-assisted in situ keratomileusis (FS-LASIK)

**Fig. 2:**
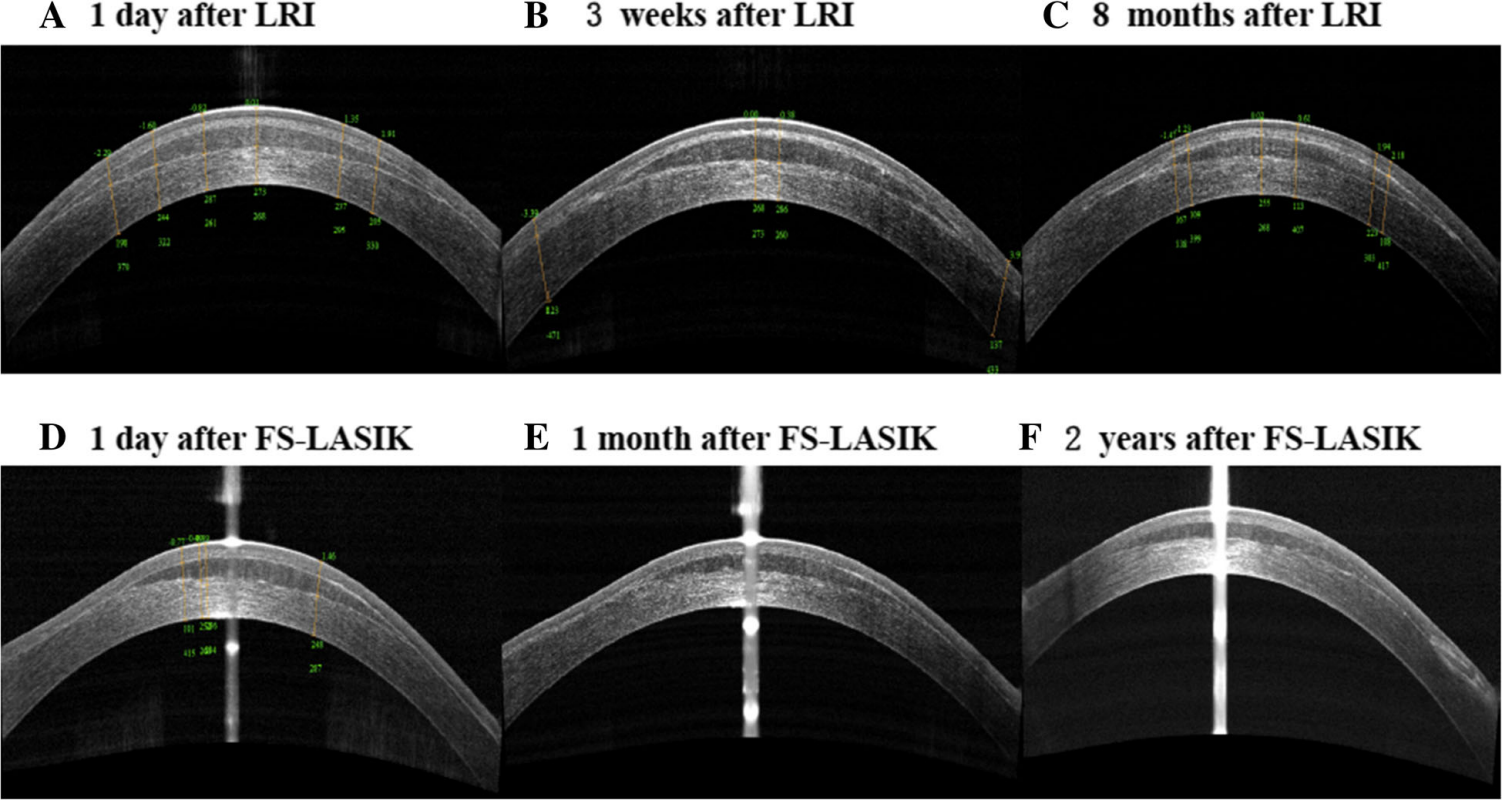
Anterior segment optical coherence tomography (OCT) images after refractive lenticule re-implantation (LRI) and after FS-LASIK

The patient was followed up at 1 day, 3 weeks, 3 months, and 8 months postoperatively. On the first day after surgery, both eyes displayed mild edema under slit-lamp observation. Corneal topography assessments revealed CCT of 520 μm and mean keratometry (K) value of 45.1 D in the left eye (Fig. 1b). Anterior optical coherence tomography (OCT) (RTVue, Optovue, Fremont, CA, USA) examination demonstrated that the re-implanted lenticule in the left eye was well attached to the stromal bed with visible demarcation lines (Fig. 2a). No displacement or striae were observed. At 8 months after lenticule in situ implantation, the refraction had remained stable since 3 months and indicated reduction of hyperopia compared with preoperative level (+ 5.00 DS/− 1.25 DC × 100°) with a stable CDVA of 20/50. The lenticule remained smoothly spread in the interface and identifiable with visible demarcation lines that showed partial hyper-reflection (Fig. 2c). The CCT was 502 μm and mean keratometry value was 43.8 D (Fig. 1d).

Due to contact lens and glasses intolerance, poor visual acuity, and severe anisometropia, the patient asked for retreatment. After the risks, benefits, and alternatives had been explained and the patient had provided informed consent, he underwent standard FS-LASIK on the left eye to correct hyperopia and astigmatism 8 months after lenticule re-implantation. A small treatment suction cone was applied. The flap had a diameter of 7.9 mm, thickness of 90 μm, standard 90° hinges, and 90° side cut angles. Refractive correction was + 5.0 DS/− 0.75 DC × 100° with attempted ablation thickness of 90 μm, optical zone of 6.5 mm, and transition zone of 1.5 mm with blend to 8 mm. A MEL 80 excimer laser (Carl Zeiss Meditec) was used for stromal ablation with a pulse energy of 185 nJ, followed by flap reposition. A silicone hydrogel contact lens was applied as a bandage, in order to avoid postoperative flap displacement or microdistortion, then removed the next day.

At postoperative day 1, the cornea exhibited mild edema and the lenticule was spread smoothly on the stromal bed with decreased peripheral thickness (Fig. 2d). Corneal topography assessments revealed a CCT of 477 μm and mean keratometry value of 51.3 D. The UDVA, manifest refraction, CDVA, mean keratometry, CCT, and corneal volume (as measured by Pentacam) before operation, after refractive lenticule re-implantation, and after LASIK are displayed in Table 1. Figures 1 and 2 show the patient’s assessments over the postoperative course. During 2 years of follow-up, the cornea was transparent with no flap striae, inflammation, epithelial ingrowth, or diffuse lamellar keratitis under slit-lamp examination. Corneal tomography showed no signs of ectasia. The UDVA, manifest refraction, and CDVA at the last visit were 20/63, − 0.75 DS/− 0.25 DC × 165°, and 20/50 in the left eye, whereas they were 20/40, + 0.5 DS/− 0.5 DC × 150°, and 20/32 in the right eye. The patient gained binocular uncorrected distance visual acuity of 20/40 and near visual acuity of 20/50, achieving improved satisfaction.

**Table 1:** The data of Patient’s left eye before operation and after refractive lenticule re-implantation and after FS-LASIK

Parameter	Preoperative	Postoperative						
		After refractive lenticule re-implantation				After FS-LASIK		
		1 day	3 weeks	3 months	8 months	1 day	1 month	2 years
UDVA	20/125	20/125	20/125	20/200	20/200	20/100	20/100	20/63
Manifest refraction (D)	+ 7.75DS/− 1.25 DC × 5°	/	+ 6.00DS/− 1.00 DC × 100°	+ 5.00DS/− 1.25 DC × 100°	+ 5.00DS/− 1.25 DC × 100°	−1.50DS/− 0.25 DC × 30°	−1.25DS/− 1.25 DC × 120°	−0.75DS/− 0.25 DC × 165°
CDVA	20/50	/	20/50	20/50	20/50	20/63	20/50	20/50
CCT (μm)	517	520	518	512	502	477	452	447
mean K (D)	41.4	45.1	43.6	43.4	43.8	51.3	50.1	49.8
Cornea Volume (mm^3^)	57.2	55.6	55.5	54.9	53.7	51.1	51.1	50.6

*UDVA* uncorrected distance visual acuity, *D* diopters, *CDVA* corrected distance visual acuity, *CCT* central corneal thickness, *K* keratometry, *DS* diopters sphere, *DC* diopters cylinder

## Discussion and conclusions

The SMILE procedure provides similar results to other corneal refractive procedures for correction of myopia, in terms of safety, efficacy, predictability, accuracy, and stability; moreover, it is flap-free and involves minimal trauma [2, 3]. However, intraoperative and postoperative complications have been reported [1, 11]. To the best of our knowledge, we report the first case of a mistaken SMILE procedure, in which myopic refractive lenticule in situ implantation successfully reversed the surgery, thereby remedying medical negligence; residual refractive error was corrected by FS-LASIK following lenticule re-implantation.

It was quite difficult to appropriately manage this case. The first step was to re-implant the lenticule in situ and maintain the original structure of the cornea. Notably, it was difficult to identify the anterior and posterior surfaces of the lenticule after extraction, because there were no marks. Fortunately, the lenticule was successfully re-implanted with the proper orientation and there were no abnormalities, such as infection or striae after re-implantation; CDVA did not decline after 3 weeks. If the lenticule had been placed in the incorrect orientation, we presume that the corneal stoma would have shown structural remodeling under intraocular pressure, leading to changes in both refractive error and corneal curvature. The resultant refractive error could have been corrected by FS-LASIK after refractive status had stabilized.

Corneal topography examinations and slit-lamp microscope observation showed mild edema that gradually improved. At 8 months after lenticule re-implantation, CCT and cornea volume were 502 μm and 53.7 mm^3^, near the preoperative values of 517 μm and 57.2 mm^3^. This has been confirmed by previous studies, which demonstrated that corneal thickness and volume could be restored to preoperative levels after post-SMILE autologous lenticule re-implantation [4, 6]. Interestingly, the mean K value increased from 41.4 D preoperatively to 45.1 D on postoperative day 1, then decreased to 43.8 D at the 8-month follow-up; spherical equivalent refraction decreased from + 7.13 DS before surgery to + 4.38 DS and remained stable within 8 months after lenticule re-implantation, showing reduction of hyperopia. Thus, anterior corneal curvature may contribute to prediction of refractive status. Pradhan et al. [5] described a patient who underwent implantation of an allogeneic lenticule obtained from a − 10.5 D myopic donor via SMILE for the correction of high hyperopia, but achieved only 50% of the intended correction at the 1-year follow-up. Similarly, Ganesh et al. [10] reported undercorrection of transplanted corneal lenticules for hyperopia after cryopreservation. Sun et al. [7] demonstrated that autologous lenticule transplantation was feasible for treatment of hyperopia in patients with anisometropia; however, refractive predictability was unsatisfactory, which may have been related to the shape of lenticule following astigmatism correction. We speculate that corneal edema in the early stage, epithelial remodeling, anterior and posterior surface changes, and postoperative wound healing contributed to the changes in this case. Moreover, refractive predictability after lenticule re-implantation requires further investigation.

After refraction became stable following lenticule re-implantation, standard FS-LASIK was performed. A thin flap of 90 μm was created to avoid cutting the flap on the re-implanted lenticule or inducing lenticule dislocation after the flap was lifted. Hyperopic ablation reached the residual stroma of the 110-μm cap; however, we did not expect this to affect refractive outcome, because the ablation would not induce displacement of the re-implanted lenticule. Due to the accuracy of femtosecond laser and smooth scanning, the patient achieved a satisfactory outcome after FS-LASIK treatment. The re-implanted lenticule attached well on the stromal bed postoperatively during the 2-year follow-up, without complications. Lim et al. [12] demonstrated the feasibility of LASIK after reversal of myopic SMILE through refractive lenticule re-implantation in a rabbit model. Our results suggest that SMILE lenticule re-implantation to restore corneal volume and thickness in a human cornea, followed by LASIK to correct hyperopia and astigmatism is feasible. Although lenticule re-implantation resulted in unpredictable refractive outcome, FS-LASIK was able to correct the residual refractive error. Nevertheless, alternative methods could have been used in this case, including a CIRCLE procedure with the VisuaMax femtosecond laser and conversion of the cap into a flap followed by excimer ablation on the lenticule surface. In addition, Moshirfar et al. [13] have proposed modified small incision lenticule intrastromal keratoplasty for the correction of high hyperopia.

Although we treated this case successfully, the cause of this medical negligence should be considered. This case emphasizes the importance of preoperative surgical checks. It is necessary to verify the patient’s information before and during surgery, in order to minimize the risk of unintentional events. Moreover, surgeons must remain calm following rare complications or medical negligence during refractive surgery.

Refractive lenticule in situ implantation offers a solution for corneal volume and thickness restoration during SMILE, which is a reversible procedure; however, refractive predictability requires further investigation. The findings in this report indicate that FS-LASIK provides feasible correction of residual refractive error following lenticule re-implantation. Future studies are needed for determining the effectiveness of the treatment.

## Data Availability

All data generated or analyzed during this study are included in this article.

## Abbreviations

BSS: Balanced salt solution
CCT: Central corneal thickness
CDVA: Corrected distance visual acuity
DC: Diopters cylinder
DS: Diopters sphere
FS-LASIK: Femtosecond laser-assisted in situ keratomileusis
LASIK: Laser-assisted in situ keratomileusis
OCT: Optical coherence tomography
SMILE: Small incision lenticule extraction
UDVA: Uncorrected distance visual acuity
